# What is the accuracy, sensitivity and specificity of the radiological peritoneal cancer index in repeat cytoreductive surgery: a retrospective study

**DOI:** 10.1186/s12957-025-03775-5

**Published:** 2025-04-11

**Authors:** Celine Garrett, Louise Sun, Raymond Hayler, Ruwanthi Wijayawardana, Nima Ahmadi, Mina Sarofim, David L. Morris

**Affiliations:** 1 Liver and Peritonectomy Unit, St George Hospital, Gray Street, Kogarah, NSW 2217 Australia; 2 Faculty of Medicine & Health, St George and Sutherland Clinical School (University of New South Wales), St George Hospital, Clinical Sciences (WRPitney) Building, Short Street, Kogarah, NSW 2217 Australia

**Keywords:** Repeat cytoreductive surgery and hyperthermic intraperitoneal chemotherapy, Peritoneal cancer index, Computed tomography, Positron emission topography, Magnetic resonance imaging

## Abstract

**Background:**

Repeat cytoreductive surgery (CRS) and hyperthermic intraperitoneal chemotherapy (HIPEC) (rCRS-HIPEC) has improved the long-term survival of select patients with acceptable perioperative morbidity and mortality. The pattern of peritoneal disease recurrence is critical in determining eligibility for rCRS-HIPEC. This study evaluated the accuracy, sensitivity and specificity of the radiological peritoneal cancer index (PCI) across different imaging modalities in rCRS-HIPEC patients.

**Methods:**

This was a retrospective study on patients with peritoneal disease recurrence who underwent rCRS-HIPEC between January 2022 to December 2023. The accuracy, sensitivity, and specificity of the radiological PCI in predicting the surgical PCI was calculated overall and for each imaging modality at each abdominal region.

**Results:**

32 patients were included in this study. The accuracy, sensitivity and specificity of the overall radiological PCI was 63.0%, 30.8% and 79.9%, respectively. Accuracy (67.5 vs. 62.6%) and specificity (84.8% vs. 75.8%) were higher in FDG-PET versus CT. The sensitivities of all imaging modalities were low (CT 34.9%, FDG-PET 33.3%). FDG-PET and CT had high sensitivities in detecting pelvic disease (80% and 87.5%) but low sensitivities in identifying small bowel (25-33.3% for both modalities) and epigastric disease (25% and 0%). For each abdominal region, the difference between radiological and surgical PCI did not differ significantly based on imaging modality.

**Conclusions:**

Overall, the radiological PCI has a good specificity in rCRS-HIPEC patients and should be used to guide perioperative decision-making. FDG-PET had superior accuracy and specificity in comparison to CT in detecting peritoneal disease recurrence.

## Background

Peritoneal carcinomatosis is the presence of malignant cells within the peritoneal layers of the abdomen and can be either from primary peritoneal cancers (e.g. mesothelioma) or from the dissemination of disease from other sites. For example, synchronous peritoneal metastases are present in up to 61% of ovarian tumours, 4.3% of colorectal cancers and 40% of gastric cancers [1–3]. The peritoneal cancer index (PCI), developed by Sugarbaker in 1990, is the most widely validated tool that quantifies the extent of peritoneal disease in a standardised fashion by dividing the abdominal cavity into 13 regions [4]. Most importantly, it is used to select and prognosticate patients undergoing cytoreductive surgery (CRS) with or without hyperthermic intraperitoneal chemotherapy (HIPEC). At present, a surgical evaluation of the PCI is the gold standard, typically done during a preoperative staging laparoscopy in preparation for CRS and HIPEC.

Evaluation of PCI may also be done less-invasively based on radiological investigations. Computed tomography (CT), magnetic resonance imaging (MRI) and positron emission tomography (PET) are the most commonly used modalities. However, their ability to determine peritoneal disease may vary due to the size and location of nodules, the underlying tumour biology, the presence of motion artefact and concurrent inflammation [5–7]. Studies have demonstrated superior diagnostic accuracy of FDG-PET in comparison to CT in identifying peritoneal disease, mesenteric disease and subdiaphragmatic involvement in those who have non-mucinous tumours [8, 9].

In a select cohort of patients who develop recurrent peritoneal disease, repeat CRS and HIPEC (rCRS-HIPEC) have been shown to confer improved long-term survival with acceptable perioperative morbidity and mortality [10, 11]. However, patient selection is critical. Having a long disease-free interval (ideally > two years which is indicative of favourable tumour biology), either no or oligometastatic extra-abdominal disease, a low burden of peritoneal disease amenable to surgical resection, a complete cytoreduction at primary CRS and HIPEC, few medical comorbidities and a good functional status comprises the inclusion criteria for rCRS-HIPEC [12]. In patients undergoing rCRS-HIPEC, an accurate evaluation of PCI by staging laparoscopy is often precluded and arguably impossible due to extensive adhesions following initial CRS. As such, greater value is placed on the radiological PCI to guide patient selection for rCRS-HIPEC. Current literature has evaluated the accuracy of radiological PCI for primary CRS and HIPEC procedures, however, there is no evaluation of its precision in rCRS-HIPEC. This study’s primary aim is to evaluate the accuracy, sensitivity, and specificity of the overall radiological PCI in peritoneal malignancy patients undergoing rCRS-HIPEC. The secondary aim is to compare the accuracy, sensitivity and specificity of different imaging modalities in determining the overall radiological PCI and the PCI of each abdominal region.

## Methods

### Study design

A retrospective study on patients treated at the Peritonectomy Unit, St George Hospital, Sydney, Australia between January 2022 to December 2023 (inclusive) was conducted. This study was designed to align with the Strengthening the Reporting of Observational Studies in Epidemiology (STROBE) guidelines [13].

### Participants

The inclusion criteria were adult patients (aged 18 years or older) with either endoscopic, pathological or radiological diagnosis of recurrence of their peritoneal malignancy who underwent rCRS-HIPEC. Patients were excluded if there was inadequate documentation of their radiological PCI at the preoperative multidisciplinary team meeting or their surgical PCI in the rCRS-HIPEC operation report. The research related to human use has complied with all the relevant national regulations, institutional policies, and in accordance with the tenets of the Helsinki Declaration, and has been approved by the South Eastern Sydney Local Health District Human Research Ethics Committee as part of “Clinical studies in Abdominal and Peritoneal Cancers”, QAQI/18/078.

### Variables

**Fig. 1:**
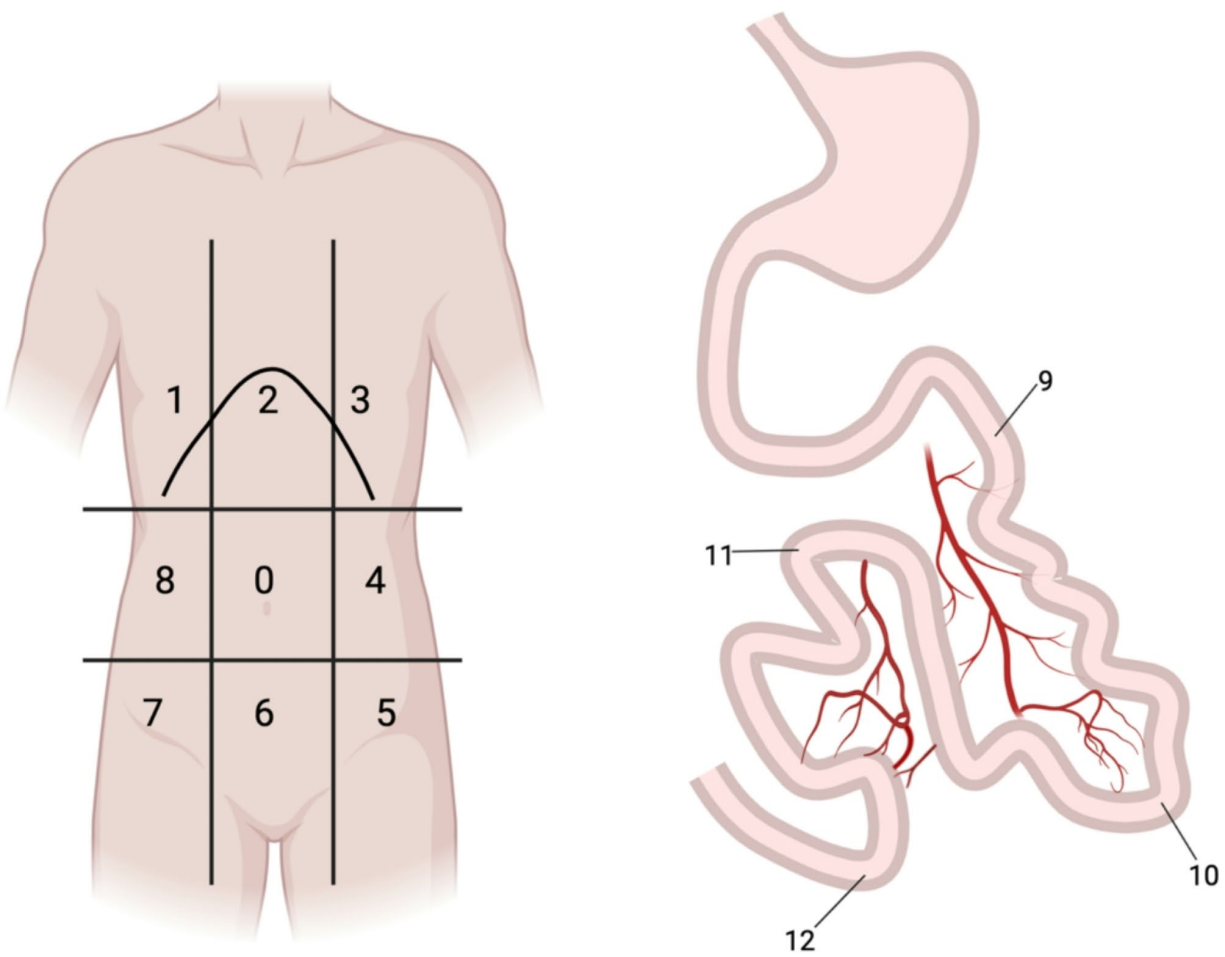
The division of the abdomen into thirteen regions as part of the PCI score

The abdomen was divided into 13 regions (Fig. 1). The PCI was calculated by giving a score to the largest tumour in each region based on its size (0 = no tumour, 1 = tumour < 0.5 cm, 2 = tumour 0.5–5 cm, 3 = tumour > 5 cm). The total PCI was the sum of the scores from each region, with a maximum score of 39.

### Data sources

All demographic, radiological and operative data was obtained via patients’ electronic medical records. Demographic data included gender, age and primary peritoneal tumour. Radiological data included the radiological PCI (total and by each region) and imaging modality (CT, FDG-PET or MRI) which was obtained via reviewing the preoperative multidisciplinary team minutes. Operative data included the surgical PCI (total and by each region) which was included in the operation reports, the number of CRS and HIPEC operations and the time interval from the most recent CRS and HIPEC.

### Radiological PCI

All radiological PCIs were determined after meticulous review of the imaging by an experienced surgical oncology board, comprising of three peritonectomy surgeons and two specialist radiologists as part of a preoperative multidisciplinary team meeting. Peritoneal recurrence was based on the presence of soft tissue abnormalities on imaging. When available, this was correlated with an FDG-PET (if the patient had previously FDG-avid disease), other imaging, endoscopic findings, clinical symptoms and tumour markers. The same team of surgeons and radiologists attended each weekly meeting over the course of this study period which minimised any variance in PCI interpretation. Specific scanning parameters were not controlled for in this study as a large proportion of patients had their imaging performed in an outpatient setting in private radiology centres.

### Surgical PCI

All patients underwent CRS according to the principles established by Sugarbaker [14]. Following a midline incision from the xiphisternum to the pubic symphysis and adhesiolysis, all 13 regions of the peritoneal cavity were examined to obtain the surgical PCI. Where fibrotic adhesions could not be differentiated from malignant peritoneal nodules, a frozen section was sent for intra-operative histopathological review.

### Statistical methods

IBM SPSS Statistics version 29 was used for all statistical analyses. The Shapiro-Wilk test for normality determined this dataset to be non-parametric. Continuous variables were presented as medians (interquartile (IQR)) and categorical variables were presented as percentages (%). The (binary) presence or absence of disease in each abdominal region (as reported by the surgical PCI) determined the true positivity/negativity and false positivity/negativity of the radiological PCI. For example, if the preoperative PCI indicated disease in region 1 but intraoperatively no peritoneal disease was found in region 1, then this was deemed to be a false positive. For each abdominal region, the accuracy, sensitivity, and specificity of the radiological PCI was calculated. These were then averaged to provide the accuracy, sensitivity, and specificity of the radiological PCI in its entirety. This was performed for each imaging modality except for MRI due to its smallest sample size (*n* = 4). The Mann-Whitney U test was used to evaluate if the total radiological and surgical PCI differed significantly. The Kruskal-Wallis H test was performed to ascertain if the difference in radiological and surgical PCI scores was affected by imaging modality for each anatomical region of the abdomen.

## Results

### Descriptive data

A total of 32 patients underwent rCRS-HIPEC during the study period and were included. Pseudomyxoma Peritonei (PMP) (*n* = 20, 62.5%,) was the most common type of tumour biology, followed by colorectal adenocarcinoma (*n* = 7, 21.7%), ovarian carcinoma (6.3%, *n* = 2), mesothelioma (*n* = 2, 6.3%) and adrenocortical carcinoma (*n* = 1, 3.1%). Whilst most patients had undergone only one previous CRS and HIPEC (*n* = 17, 53.1%), the maximum number of prior CRS and HIPEC operations was four. The median operative interval from patients’ previous CRS and HIPEC was 21.5 (IQR: 11.3–46.5) months. CT was used to assess the radiological PCI in 15 patients (46.9%), an FDG-PET in 13 patients (40.6%) and MRI in four patients (12.5%).

The accuracy, sensitivity and specificity of the overall radiological PCI (as determined by any imaging modality).

The accuracy of the radiological PCI for all 13 abdominal regions was 63.0%. The sensitivity and specificity were 30.8% and 79.9%, respectively. Across the entire cohort, the median radiological and surgical PCIs were 6.5 (IQR: 3.3–12.0) and 6.5 (IQR: 3.0-18.8), respectively which did not significantly differ. The radiological PCI underestimated the surgical PCI in 50% of the cohort (*n* = 16), overestimated it in 40.6% (*n* = 13) and correctly predicted it in 9.4% (*n* = 3).

The accuracy, sensitivity and specificity of CT and FDG-PET in determining the overall PCI and for each anatomical region.

FDG-PET was more accurate (67.5% vs. 62.6%) and specific (84.8% vs. 75.8%) than CT at determining the radiological PCI across all abdominal regions. The sensitivities of FDG-PET and CT imaging modalities were low (33.3 and 34.9%, respectively). The performance parameters of each imaging modality for individual anatomical regions of the abdomen are demonstrated in Table 1. When each abdominal region was considered independently, each imaging modality showed non-significant differences between radiological and surgical PCI.

**Table 1:** The accuracy, sensitivity and specificity of CT, FDG-PET and MRI abdomen at 13 anatomical regions

Region		TN	TP	FN	FP	Accuracy	Sensitivity	Specificity
**0**								
	CT CAP	9	3	1	2	75.0%	58.3%	85.0%
	FDG-PET	6	3	3	1	80.0%	75.0%	81.8%
	MRI abdomen	2	1	1	0	75.0%	50.0%	100.0%
**1**								
	CT CAP	6	2	2	5	53.3%	50.0%	54.5%
	FDG-PET	11	0	2	0	84.6%	0.0%	100.0%
	MRI abdomen	2	0	2	0	50.0%	0.0%	100.0%
**2**								
	CT CAP	8	1	3	3	60.0%	25.0%	72.7%
	FDG-PET	7	0	5	1	53.8%	0.0%	87.5%
	MRI abdomen	3	0	1	0	75.0%	0.0%	100.0%
**3**								
	CT CAP	9	2	0	4	73.3%	100.0%	69.2%
	FDG-PET	6	3	0	4	69.2%	100.0%	60.0%
	MRI abdomen	1	0	2	1	25.0%	0.0%	50.0%
**4**								
	CT CAP	9	2	3	1	73.3%	40.0%	90.0%
	FDG-PET	9	1	3	0	76.9%	25.0%	100.0%
	MRI abdomen	2	0	2	0	50.0%	0.0%	100.0%
**5**								
	CT CAP	8	2	5	0	66.7%	28.6%	100.0%
	FDG-PET	6	1	5	1	53.8%	16.7%	85.7%
	MRI abdomen	3	0	1	0	75.0%	0.0%	100.0%
**6**								
	CT CAP	4	4	1	6	53.3%	80.0%	40.0%
	FDG-PET	3	7	1	2	76.9%	87.5%	60.0%
	MRI abdomen	0	2	0	2	50.0%	100.0%	0.0%
**7**								
	CT CAP	6	1	7	1	46.7%	12.5%	85.7%
	FDG-PET	5	2	4	2	53.8%	33.3%	71.4%
	MRI abdomen	2	0	2	0	50.0%	0.0%	100.0%
**8**								
	CT CAP	7	1	7	0	53.3%	12.5%	100.0%
	FDG-PET	11	0	2	0	84.6%	0.0%	100.0%
	MRI abdomen	2	0	2	0	50.0%	0.0%	100.0%
**9**								
	CT CAP	12	1	2	0	86.7%	33.3%	100.0%
	FDG-PET	10	0	3	0	76.9%	0.0%	100.0%
	MRI abdomen	2	0	2	0	50.0%	0.0%	100.0%
**10**								
	CT CAP	8	1	5	1	60.0%	16.7%	88.9%
	FDG-PET	8	0	5	0	61.5%	0.0%	100.0%
	MRI abdomen	1	0	1	2	25.0%	0.0%	33.3%
**11**								
	CT CAP	9	1	3	2	66.7%	25.0%	81.8%
	FDG-PET	8	1	3	1	69.2%	25.0%	88.9%
	MRI abdomen	1	0	2	1	25.0%	0.0%	50.0%
**12**								
	CT CAP	5	1	2	7	40.0%	33.3%	41.7%
	FDG-PET	5	1	2	5	46.2%	33.3%	50.0%
	MRI abdomen	2	0	2	0	50.0%	0.0%	100.0%
**Totals**								
	CT CAP	100	22	41	32	62.6%	34.9%	75.8%
	FDG-PET	95	19	38	17	67.5%	33.3%	84.8%
	MRI abdomen	23	3	20	6	50.0%	13.0%	79.3%

**Table 2:** The accuracy, sensitivity and specificity of CT versus FDG-PET in detecting the radiological PCI for each anatomical region of the abdomen

Region	Modality	TN	TP	FN	FP	Accuracy	Sensitivity	Specificity
**0**	CT CAP	9	3	1	2	75.0%	58.3%	85.0%
	FDG-PET	6	3	3	1	80.0%	75.0%	81.8%
**1**	CT CAP	6	2	2	5	53.3%	50.0%	54.5%
	FDG-PET	11	0	2	0	84.6%	0.0%	100.0%
**2**	CT CAP	8	1	3	3	60.0%	25.0%	72.7%
	FDG-PET	7	0	5	1	53.8%	0.0%	87.5%
**3**	CT CAP	9	2	0	4	73.3%	100.0%	69.2%
	FDG-PET	6	3	0	4	69.2%	100.0%	60.0%
**4**	CT CAP	9	2	3	1	73.3%	40.0%	90.0%
	FDG-PET	9	1	3	0	76.9%	25.0%	100.0%
**5**	CT CAP	8	2	5	0	66.7%	28.6%	100.0%
	FDG-PET	6	1	5	1	53.8%	16.7%	85.7%
**6**	CT CAP	4	4	1	6	53.3%	80.0%	40.0%
	FDG-PET	3	7	1	2	76.9%	87.5%	60.0%
**7**	CT CAP	6	1	7	1	46.7%	12.5%	85.7%
	FDG-PET	5	2	4	2	53.8%	33.3%	71.4%
**8**	CT CAP	7	1	7	0	53.3%	12.5%	100.0%
	FDG-PET	11	0	2	0	84.6%	0.0%	100.0%
**9**	CT CAP	12	1	2	0	86.7%	33.3%	100.0%
	FDG-PET	10	0	3	0	76.9%	0.0%	100.0%
**10**	CT CAP	8	1	5	1	60.0%	16.7%	88.9%
	FDG-PET	8	0	5	0	61.5%	0.0%	100.0%
**11**	CT CAP	9	1	3	2	66.7%	25.0%	81.8%
	FDG-PET	8	1	3	1	69.2%	25.0%	88.9%
**12**	CT CAP	5	1	2	7	40.0%	33.3%	41.7%
	FDG-PET	5	1	2	5	46.2%	33.3%	50.0%
**Overall**	CT CAP	100	22	41	32	62.6%	34.9%	75.8%
	FDG-PET	95	19	38	17	67.5%	33.3%	84.8%

## Discussion

Despite the efficacy of primary CRS and HIPEC, 31–57% of patients will have isolated peritoneal recurrence of their disease [12, 15, 16]. Over the last decade, the feasibility and survival benefit of rCRS-HIPEC has been demonstrated in select patients. Sarofim et al. [17] conducted a systematic review of rCRS-HIPEC for colorectal peritoneal metastases and found a 16.7–37.5% morbidity rate and 0% mortality rate which is comparable to primary CRS and HIPEC. Choudry et al. [18] analysed 1294 patients with a variety of primary cancer types and found that overall survival was significantly better in patients undergoing rCRS-HIPEC (*n* = 125) in comparison to those who did not (104 vs. 55 months, *p* < 0.0010). In an analysis by Karpes et al. [11] of 462 patients with appendiceal tumours, 102 underwent rCRS-HIPEC which conferred a survival benefit for patients with high-grade tumours (90.7 vs. 55.6 months, *p* = 0.016). Ahmadi et al. [10] analysed 430 PMP patients with recurrence and showed that 5-year overall survival was superior in patients who underwent rCRS with or without HIPEC (*n* = 85), followed by the “watch and wait” approach (*n* = 119), maximal tumour debulking and palliative chemotherapy (*n* = 119) (89.6% vs. 77.4% vs. 62.2% vs. 22.8%, *p* < 0.001). The most common primary site of cancer in these studies was appendiceal, followed by colorectal, mesothelioma and ovarian which is consistent with our study.

Since the selection of patients for rCRS-HIPEC is highly dependent on the volume and location of disease recurrence, the radiological PCI is of utmost importance. However, no other studies have investigated the accuracy, sensitivity, and specificity of the radiological PCI in rCRS-HIPEC. Thus, the findings of this study are novel. The accuracy and sensitivity of the overall radiological PCI in this study were 63.0% and 30.8%, respectively. The accuracy was within the reported range for primary CRS and HIPEC (30–88%), but the sensitivity was lower (55–76%) [6, 19–23]. Sensitivity also remained low for all imaging modalities in our study. The specificity of the radiological PCI in rCRS-HIPEC was 79.9% which was consistent with other documented values for primary CRS and HIPEC (69-95.1%) [20, 24]. This highlights the value of the radiological PCI in the surveillance of CRS and HIPEC patients (for example, if the radiological PCI is zero and there is no concern for recurrence, then one can be reassured that the likelihood of peritoneal disease recurrence is low). However, when the radiological PCI is zero but the patient has worrying symptoms and elevated tumour markers, then the surgeon should have a low threshold for suspicion of peritoneal disease recurrence and the patient must be discussed at a multidisciplinary team meeting.

Although not significant, FDG-PET better determined the overall radiological PCI in rCRS-HIPEC patients in comparison to CT scans. Specifically, FDG-PET had an accuracy and specificity of 67.5% and 84.8%, respectively. This finding is due to the ability of FDG-PET to differentiate metabolically and functionally active lesions from those that are not. Despite its apparent superiority in our rCRS-HIPEC cohort, this is lower than reported values for primary CRS and HIPEC patients in the literature [25]. A large portion of patients eligible for rCRS-HIPEC will have tumours with mucinous histology [26]. However, the presence of high-volume acellular mucin has limited metabolic activity and is thus not FDG-PET-avid [27, 28]. Unfortunately, the presence of mucin was not included as a data variable in this study and thus the correlation between mucinous tumours and the performance of different imaging modalities was not conducted. The accuracy, sensitivity, and specificity of CT scans in determining the overall radiological PCI is extremely variable ranging from 40 to 100% in papers evaluating primary CRS and HIPEC patients [29]. In our rCRS-HIPEC cohort, its accuracy, sensitivity, and specificity were 62.6%, 75.8% and 34.9%. The variability of imaging modalities in this study demonstrates the need for future research to develop surveillance imaging protocols specific to peritoneal disease and to evaluate the cost-effectiveness of routine FDG-PETs. Further, when there is a clinical or pathological concern for peritoneal recurrence and a normal CT, an FDG-PET should be the next line of investigation.

The pattern of intraperitoneal disease recurrence following CRS and HIPEC impacts a patient’s eligibility for rCRS-HIPEC. For example, hard and infiltrative recurrence involving a substantial amount of small bowel is a poor prognostic factor that will preclude a patient from rCRS-HIPEC [12]. In prior studies, the abdominal region has impacted the performance of different imaging modalities in detecting peritoneal disease [22, 30–34]. In our study, all imaging modalities had a sensitivity of over 80% in detecting pelvic recurrence (region 6). However, all imaging modalities had a low sensitivity in the detection of small bowel disease (regions 9–12). The difficulty in determining small bowel disease has previously been attributed to small nodule size (< 1 cm) and a “layered-type” of peritoneal carcinomatosis where the small bowel is coated by thin cancerous plaques that manifest as wall thickening and distortion which can be missed when small bowel loops are collapsed [31, 35]. Further, metabolic activity in the small bowel causes physiological FDG uptake on a PET scan. The level of this uptake may be influenced by bowel motility, reactive lymphocytes and recent food intake, thus making the distinction of peritoneal disease challenging. Detection of peritoneal disease in the stomach/less sac (region 2) was also generally poor with CT having a sensitivity of 50% but FDG-PET having a sensitivity of 0%. This may be explained by the anatomical complexity of the lesser sac due to its many anatomical relations, multiple recesses and relatively small size. As such, CT enterography (which uses neutral oral contrast to distend the bowel) and endoscopic ultrasound may be suitable adjuncts to assess radiological PCI in rCRS-HIPEC patients, however, data is limited and future research is warranted [36, 37].

Although this study is the first to report radiological accuracy in rCRS, we acknowledge important limitations. Firstly, this is a retrospective study and thus selection bias may be present. Secondly, the accuracy, sensitivity and specificity were calculated based on the binary presence or absence of disease in each abdominal region. Therefore, this study was unable to determine the quantitative discrepancies between the radiological and surgical PCI scores. Secondly, the cohort consisted only of patients undergoing rCRS and thus comparison with radiological PCI in primary CRS patients was only performed based on the available literature. A future study including both cohorts of patients should be completed. Thirdly, the cohort size was small in this study, however, it was performed at the highest volume CRS and HIPEC centre in the Southern Hemisphere, and the sample size is limited by the highly selective nature of rCRS-HIPEC itself. Thus, future research should be multi-institutional. Fourthly, the performance of MRI in determining the radiological PCI of rCRS-HIPEC patients was not performed due to a small sample size (*n* = 4). At our centre, MRI scans are not performed routinely to ascertain the radiological PCI and are only executed in patients with suspected liver metastases, pelvic tumours, or severe allergies to intravenous contrast. This is due to its limited availability and out-of-pocket cost. Finally, radiological variables such as lesion size, mucinous component, and the presence of ascites, may have added strength to the data as well as a correlation between radiological, surgical and pathological PCI and survival.

## Conclusions

The increasing proportion of patients undergoing rCRS-HIPEC requires accurate preoperative radiological assessment of PCI to carefully select patients who will achieve a survival benefit. The radiological PCI (obtained from any imaging modality) had a high specificity, moderate accuracy and low sensitivity in predicting the surgical PCI in rCRS-HIPEC patients. Further, FDG-PET was preferable to CT in evaluating the radiological PCI due to its higher accuracy and specificity. For each abdominal region, the difference between the radiological and surgical PCI did not differ significantly based on imaging modality. All imaging modalities performed well in identifying disease in the pelvis but not in the small bowel and upper mid-abdomen.

## Data Availability

The datasets generated during and/or analysed during the current study are available from the corresponding author upon reasonable request.

## Abbreviations

PCI: Peritoneal cancer index
CRS: Cytoreductive surgery
HIPEC: Hyperthermic intraperitoneal chemotherapy
CT: Computed tomography
MRI: Magnetic resonance imaging
PET: Positron emission tomography
rCRS-HIPEC: Repeat CRS and HIPEC
STROBE: Strengthening the Reporting of Observational Studies in Epidemiology
IQR: Interquartile range
PMP: Pseudomyxoma peritonei
